# Molecular species identification, host preference and detection of myxoma virus in the *Anopheles maculipennis* complex (Diptera: Culicidae) in southern England, UK

**DOI:** 10.1186/s13071-015-1034-8

**Published:** 2015-08-15

**Authors:** Victor A. Brugman, Luis M. Hernández-Triana, Sean W. J. Prosser, Chris Weland, David G. Westcott, Anthony R. Fooks, Nicholas Johnson

**Affiliations:** Vector-borne viral diseases programme, The Pirbright Institute, Ash Road, Woking, GU24 0NF UK; Department of Disease Control, London School of Hygiene and Tropical Medicine, Keppel Street, London, WC1E 7HT UK; Animal and Plant Health Agency, Woodham Lane, New Haw, Addlestone, Surrey KT15 3NB UK; Biodiversity Institute of Ontario, University of Guelph, 50 Stone Road, Guelph, Ontario N1G 2W1 Canada; School of Environmental Sciences, University of Guelph, Guelph, Ontario N1G 2W1 Canada; Department of Clinical Infection, Microbiology and Immunology, University of Liverpool, Liverpool, L69 7BE UK

**Keywords:** Mosquito, *Anopheles*, Blood-meal, Host preference, DNA barcoding, Myxoma virus

## Abstract

**Background:**

Determining the host feeding patterns of mosquitoes by identifying the origin of their blood-meals is an important part of understanding the role of vector species in current and future disease transmission cycles. Collecting large numbers of blood-fed mosquitoes from the field is difficult, therefore it is important to maximise the information obtained from each specimen. This study aimed to use mosquito genome sequence to identify the species within *Anopheles maculipennis* sensu lato (*An. maculipennis* s.l.), identify the vertebrate hosts of field-caught blood-fed *An. maculipennis* s.l. , and to test for the presence of myxoma virus (*Poxviridae*, genus *Leporipoxvirus*) in specimens found to have fed on the European rabbit (*Oryctolagus cuniculus*).

**Methods:**

Blood-fed *An. maculipennis* s.l. were collected from resting sites at Elmley Nature Reserve, Kent, between June and September 2013. Hosts that *An. maculipennis* s.l. had fed on were determined by a PCR-sequencing approach based on the partial amplification of the mitochondrial *cytochrome C oxidase subunit I* gene. Mosquitoes were then identified to species by sequencing a region of the *internal transcribed spacer-2*. DNA extracts from all mosquitoes identified as having fed on rabbits were subsequently screened using PCR for the presence of myxoma virus.

**Results:**

A total of 94 blood-fed *Anopheles maculipennis* s.l. were collected, of which 43 (46 %) provided positive blood-meal identification results. Thirty-six of these specimens were identified as *Anopheles atroparvus*, which had fed on rabbit (*n* = 33, 92 %) and cattle (*n* = 3, 8 %). Seven mosquitoes were identified as *Anopheles messeae,* which had fed on cattle (*n* = 6, 86 %) and dog (*n* = 1, 14 %). Of the 33 *An. atroparvus* that contained rabbit blood, nine (27 %) were positive for myxoma virus.

**Conclusions:**

Results demonstrate that a single DNA extract from a blood-fed mosquito can be successfully used for molecular identification of members of the *An. maculipennis* complex, blood-meal identification, and for the targeted detection of a myxoma virus. This study shows that *An. atroparvus* has a strong feeding preference for both healthy and myxoma-infected rabbits, providing evidence that this species may play a significant role in the transmission of myxomatosis among wild rabbit populations in the United Kingdom (UK).

## Background

The identification of blood-meal origin in haematophagous arthropods such as mosquitoes provides important information concerning vector-host interactions and associated disease transmission dynamics [1]. Molecular techniques for blood-meal identification and the increasing volume of openly accessible databases of host species identification data such as GenBank [2] and The Barcode of Life Database [3] have facilitated this area of research. Systematic characterisation of bird and mammalian host genetics in particular has increased the specificity of studies carried out driven by the use of polymerase chain reaction techniques. These techniques have largely replaced serological methods for blood meal identification such as the precipitin test and enzyme-linked immunosorbent assays (ELISA) [4]. The high copy number and conserved nature of mitochondrial genes such as *cytochrome b* and *cytochrome c oxidase I* (COI) have made them popular targets for identification applied to host-feeding preference studies [5]. Ribosomal genes such as the *internal transcribed spacer-2* (ITS-2) are also commonly used in species identification [6]. For example, both COI and ITS-2 markers helped to identify a third member of the *An. maculipennis* complex, *An. daciae*, Linton, Nicolescu & Harbach 2004, from the previously recognised species *An. atroparvus* van Thiel 1927 and *An. messeae* Falleroni 1926 [7]. Identifying mosquitoes to species level is important in blood-feeding studies as sibling species may exhibit marked differences in feeding preferences which are likely to influence their role in patterns of disease transmission (reviewed in [8]).

Myxomatosis is a widespread disease of rabbits resulting from infection with the myxoma virus (*Poxviridae*, genus *Leporipoxvirus*). The virus causes mild disease in its South American native host species, rabbits of the genus *Sylvilagus* including the South American tapeti (*Sylvilagus braziliensis*), but in the European rabbit (*Oryctolagus cuniculus*) infection results in severe disease [9, 10]. Two years after its introduction into the UK in 1953 [11], myxomatosis was responsible for the death of up to 99 % of the British rabbit population [12] and although some resistance in natural populations has since emerged [13], the disease continues to cause deaths in wild and domestic rabbits. The primary vector of the myxoma virus in Britain is the rabbit flea, *Spilopsyllus cuniculi* [14]. Flea mouthparts become contaminated with myxoma virus when biting an infected rabbit, often directly through a lesion, and the virus can subsequently be mechanically transmitted to another host [15]. Other biting insects have also been implicated in transmission, most notably several species of mosquito including members of the Australian species complex *An. annulipes* [16]. In Australia, mosquitoes were the principle vector of myxomatosis until the introduction of *S. cuniculi* in 1969 in order to promote transmission of the disease for rabbit population control purposes [17]. However, the role of mosquitoes in transmission of myxoma virus in the UK is less clear. Although an early study following the initial outbreak of myxomatosis in domestic rabbits implicated *An. atroparvus*, the authors found no evidence that healthy rabbits were fed upon in the wild, therefore did not consider the species important for transmission cycles in wild rabbit populations [18]. Subsequent studies using direct capture from rabbits and rabbit-baited traps did provide evidence that healthy rabbits were bitten by mosquitoes, but at a relatively low frequency, particularly when alternative large mammals (such as livestock species) were in close proximity [19–21]. The myxoma virus was successfully identified from specimens of 17 out of the 34 British mosquito species in a limited number of studies (see Table 1), experimental transmission of myxomatosis was demonstrated using *An. atroparvus* [22] and exposure of healthy rabbits in the laboratory to the biting of field-caught mosquitoes resulted in infection [18, 19, 23]. Yet the apparent low biting and feeding rates of mosquitoes on healthy wild rabbits appeared to be a limitation to the involvement of mosquitoes in natural transmission cycles. In the UK mosquitoes might become infected through opportunistic feeding on infected rabbits with reduced host defensive behaviour but were unlikely to subsequently bite a healthy rabbit and transmit the virus.

**Table 1:** Reported rabbit-feeding behaviour of British mosquitoes and their association with the myxoma virus prior to this study

Mosquito species	Evidence of natural rabbit feeding	Identification of rabbit feeding through analysis of blood-meals	Wild-caught mosquitoes positive for *Myxoma* virus
*Aedes cinereus*	Yes [21, 23]^BC^, [19]^DC^	Yes [19, 20]	No
*Aedes rusticus*	Yes [19]^BC^	No	No
*Anopheles atroparvus* ^a^	No	No	Yes [18]
*Anopheles claviger*	Yes [19]^DC, BC^	Yes [19, 20]	Yes [19]
*Anopheles plumbeus*	Yes [19, 43]^DC, BC^	Yes [20, 21]	Yes [19]
*Coquillettidia richiardii*	Yes; [19, 43]^BC^	Yes [19–21]	No
*Culex pipiens s.l.*	Yes [43]^BC^	Yes [19, 20]	Yes [19]
*Culex torrentium*	Yes [43]^BC^	Yes [20]	No
*Culiseta annulata*	Yes [19, 43]^DC, BC^	Yes [20, 21]	No
*Culiseta litorea*	Yes	Yes [20]	No
*Culiseta morsitans*	Yes [43]^BC^, [19]^DC^	Yes [19, 21]	No
*Ochlerotatus annulipes*	Yes [23]^BC^	Yes [23]	Yes [23]
*Ochlerotatus cantans*	Yes [19, 23]^BC^	Yes [20, 23]	Yes [19, 23]
*Ochlerotatus detritus*	Yes [43]^DC, BC^, [19]^DC^	No	No
*Ochlerotatus dorsalis*	No	Yes [19, 20]	No
*Ochlerotatus punctor*	Yes [19, 20]^DC^, [43]^BC^	Yes [19]	No
*Ochlerotatus geniculatus*	Yes; [19]^DC, BC^	Yes [19]	No

Evidence of natural feeding provided by direct collections (^DC^) from rabbits, or rabbit-baited trap collections (^BC^)

^a^ *Anopheles atroparvus* was identified in these studies based on morphological and behavioural characteristics. *Culex pipiens* s.l. in these studies was identified morphologically and thus could include *Cx. pipiens f. pipiens* or *Cx. pipiens f. molestus*

A major limitation to mosquito blood-feeding studies lies with the difficulty in collecting large numbers of blood-fed individuals with blood-meals that are sufficiently undigested to allow successful DNA amplification [4]. This study aimed to maximise the data obtained from each blood-fed specimen by assessing whether a single DNA extract could be used for multiple purposes: firstly to identify the blood-meal origin in members of the *An. maculipennis* s.l using a cocktail of ‘universal’ barcoding primers and secondly to identify individual species of the *An. maculipennis* s.l. present in the study area to assess whether different species exhibited different feeding preferences. *An. maculipennis* s.l. was abundant at the site during the collecting visits and the dominant anopheline species captured. During a visit to Elmley, Kent, in the summer of 2013, it was also observed that rabbits were frequently present within 25 metres of areas where human biting activity of mosquitoes had been reported by farm workers. On a subsequent visit, wild rabbits with obvious facial lesions indicative of myxomatosis were observed. Therefore, a further aim of the study was to test whether blood-meal samples that originated specifically from rabbits also contained evidence of myxoma virus infection.

## Methods

### Collection of blood-fed mosquitoes

**Fig. 1:**
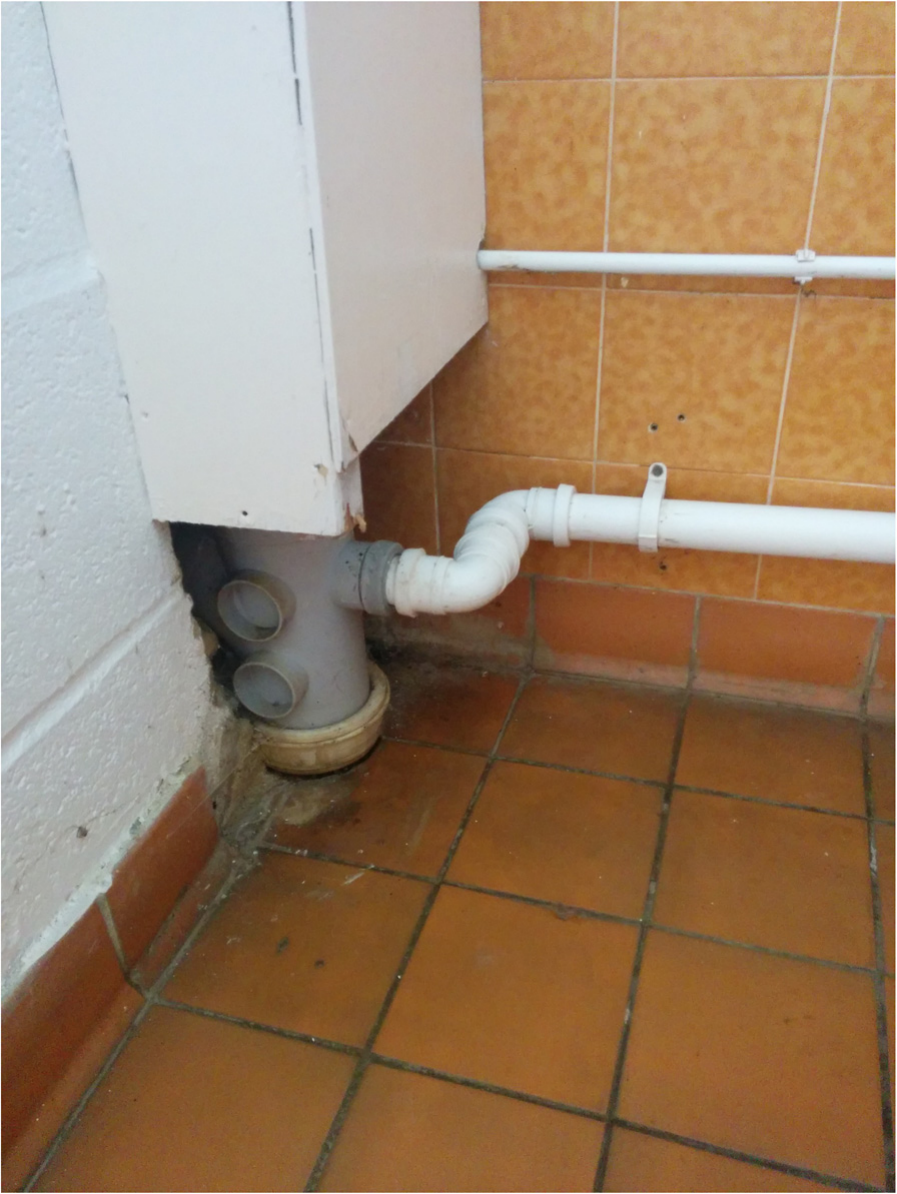
Photograph showing the primary collection area of resting mosquitoes in the toilet block at Elmley. Blood-fed *Anopheles maculipennis* s.l. mosquitoes were found resting directly on the walls and on or under the exposed wooden covering to the pipework

*An. maculipennis* s.l. were collected over 15 visits from Elmley National Nature Reserve, Isle of Sheppey (51.377445, 0.784068), Kent, UK between June and September 2013. Elmley is a freshwater coastal marsh used to graze approximately 700 head of cattle. The collection site was within 200 meters of grazing cattle. The site is popular with birdwatchers all year-round owing to the abundance of local and seasonal migrant bird species that breed in the area. Mosquitoes were primarily collected using a mouth aspirator (John W Hock, Gainsville, Florida, USA) from inside the publically accessible toilet facilities where they were observed to be resting on walls and close to exposed sections of the wood enclosing the pipework (Fig. 1). Additional attempts to collect blood-feds from a similar area were made using four CDC resting traps [24] placed in close proximity (~25 m) to the toilet block and run overnight (~14 hours) for nine of the 15 nights. Finally any anophelines landing on and attempting to feed on the collector were captured where possible. Collected mosquitoes were placed into a cooler containing dry ice and transported to the laboratory. Blood-fed specimens were separated from non-blood-fed specimens on the same day as collection and stored at −20 °C until processing at the Animal and Plant Health Agency (APHA).

### DNA extraction from mosquito abdomens

Mosquitoes were identified based on morphological features as *An. maculipennis* s.l. following the key of Snow [25]. Abdomens of engorged mosquitoes were separated from the rest of the body on a chilled plate using forceps, and placed into individual 1.5 mL eppendorf tubes containing 200 μl phosphate buffered saline (PBS). The abdomens were pressed against the wall of the tube using the forceps to release the blood-meal. The remaining head and thorax of each mosquito was stored at −20 °C for morphological reference. Forceps were cleaned between specimens using a three stage wash to avoid cross-contamination. The first wash consisted of 5 % decon, the second of 100 % ethanol and the third of sterile water, at which point all excess liquid was removed with task wipes (Kimtech Science, Roswell, Georgia, USA). Each sample was incubated with 20 μl proteinase K and 200 μl buffer AL for 30 min at 56 °C in a water bath. DNA extraction was carried out using the DNeasy Blood and Tissue Kit (QIAgen, Manchester, UK), following the manufacturer’s spin column-protocol. All DNA extractions were stored at 4 °C until processing.

### Identification of blood-meal host

Vertebrate host species in the blood-meal were identified using a vertebrate specific, M13-tailed, triple-primer cocktail (VF1_t1 + VF1d_t1 + VF1i_t1/VR1_t1 + VR1d_t1 + VD1i_t1) targeting a 685 base pair (bp) sequence of the COI gene [26, 27]. This primer combination was expected to amplify all vertebrate species. Reaction contents were obtained from Sigma-Aldrich (Sigma-Aldrich, Dorset, UK) unless otherwise stated. The final PCR reaction mix of 50 μl consisted of: 31.075 μl H_2_O, 5 μl GeneAmp 10X PCR buffer I (Applied Biosystems, Life Technologies Ltd, Paisley, UK), 1 μl dNTPs (at 0.2 mM/μl), 1 μl of each primer (at 10pmol/μl), 0.25 μl AmpliTaq Gold DNA Polymerase (10 units/μl) (Applied Biosystems, Life Technologies Ltd, Paisley, UK), 0.675 μl dimethyl sulfoxide (DMSO), 1 μl tetramethylammonium chloride (TMAC) and 5 μl extracted DNA. The thermal profile consisted of an initial denaturation step at 94 °C for 10 minutes followed by 40 cycles of: 94 °C for 30 seconds, 53 °C for 30 seconds, 72 °C for one minute, followed by a final elongation step of 72 °C for 10 minutes. PCR products were separated on a 1.5 % agarose gel and samples producing a positive result were sequenced. Sequencing was performed using M13 primers [27] at 1pmol/μl. Amplification products were sequenced in both directions using the ABI PRISM® BigDye® Terminator v3.1 Cycle Sequencing Kit (Applied Biosystems, Life Technologies Ltd, Paisley, UK). All sequences were edited using Lasergene version 12.1 (DNASTAR, Inc, Madison, Wisconsin, USA) and assigned to a particular vertebrate species when agreement was ≥98 % to sequences of known species in GenBank [28].

### Species identification within *Anopheles maculipennis* s.l.

Species level identification was obtained by amplification of a 435 bp region of ITS-2 using the primers 5.8SF and 28SR of Collins & Paskewitz [29]. PCR products were obtained using an optimised real-time PCR assay in a Mx3000P real-time PCR system (Stratagene, Agilent Technologies, Cheshire, UK) in the following reaction mix, final volume 40 μl: 2 μl of DNA template, 14 μl H_2_O, 20 μl SYBRGreen JumpStart *Taq* ReadyMix (Sigma-Aldrich, Dorset, UK) and 2 μl of each primer (each at 10 pmol/μl). The thermal profile consisted of an initial denaturation step at 94 °C for 10 minutes followed by 35 cycles of: 94 °C for 30 seconds, 53 °C for 30 seconds, 72 °C for one minute, followed by a final elongation step of 72 °C for 10 minutes. PCR products were visualized on a 1.5 % agarose gel, and samples showing bands of the correct size were sequenced in both directions using the ABI PRISM® BigDye® Terminator v3.1 Cycle Sequencing Kit (Applied Biosystems). All sequences were edited in Lasergene version 12.1 (DNASTAR) and assigned to a particular mosquito species when agreement was ≥98 % to sequences of known species in GenBank. ITS-2 sequences for each of the species within *An. maculipennis* s.l. found in the UK are available.

### Detection of myxoma virus

Myxoma virus genome was detected using two previously published methods. Samples were initially screened using Low-GC PCR primers that amplified a 220 bp sequence of the myxoma virus genome [30]. Samples giving a positive result were also amplified using a primer pair (M135Rfor/M135Rrev) [31] that produced a 650 bp amplicon. Amplified products were excised from a 2 % agarose gel and purified using a gel extraction kit (QIAgen, Manchester, UK). The resulting amplicon was then sequenced using flanking primers and ABI PRISM® BigDye® Terminator v3.1 Cycle Sequencing Kit (Applied Biosystems, Life Technologies Ltd, Paisley, UK) following the manufacturer’s instructions. The presence of myxoma virus was confirmed by BLAST (NCBI) search of the sequence.

## Results

**Fig. 2:**
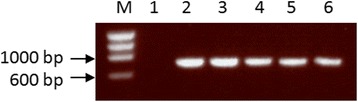
Gel image showing COI amplification products. The samples are PhiX174 DNA marker (M), negative control (1), mosquito blood-meal samples (2–4) and a positive control of DNA (5,6). The positive control was DNA extracted directly from horse blood

In total, 94 blood-fed specimens belonging to the *An. maculipennis* s.l. were collected from the Elmley site over 15 collection days. The toilet block yielded the majority (*n* = 92), with only one blood-fed *An. maculipennis* s.l. collected in the CDC resting traps and one that alighted on the collector. Of the total blood-fed samples extracted, 43 (46 %) produced a 685 bp band when amplified with COI primers as illustrated in Fig. 2. *An. maculipennis* s.l. at Elmley fed on cow (*Bos taurus*), European rabbit (*Oryctolagus cuniculus*) and dog (*Canis lupus familiaris*) (Table 2).

**Table 2:** Hosts selected by mosquito species at Elmley, Kent, between June and September 2013

Mosquito species	Cow	Rabbit	Dog	Total
*Anopheles atroparvus*	3	33	0	36
*Anopheles messeae*	6	0	1	7
Total of blood-fed	9	33	1	43

The molecular identification of the species from the same DNA samples using the ITS-2 region revealed that 36 specimens were *An. atroparvus* (98–100 % identity following BLAST search) and seven specimens were *An. messeae* (99–100 % identity following BLAST search). For the latter sequences, all shared greater than 99 % identity with published *An. messeae* ITS-2 sequences (GenBank accession number AY238412) and we are confident that no blood-fed samples of *An. daciae* were detected at the site. When the blood-feeding hosts were analyzed by species, *An. atroparvus* fed mainly on rabbits (*n* = 33, 92 %) and cattle (*n* = 3, 8 %). In contrast, *An. messeae* was found to have fed on cattle (*n* = 6, 86 %) and dog (*n* = 1, 14 %) (Table 2).

**Fig. 3:**
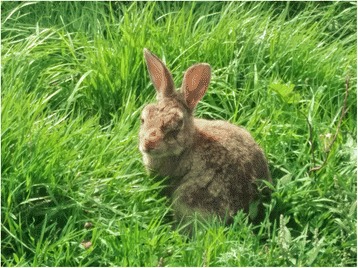
Rabbit with swelling and lesions around the eyes indicative of myxomatosis infection (photographed by VAB at Elmley, Kent in July 2014)

**Fig. 4:**
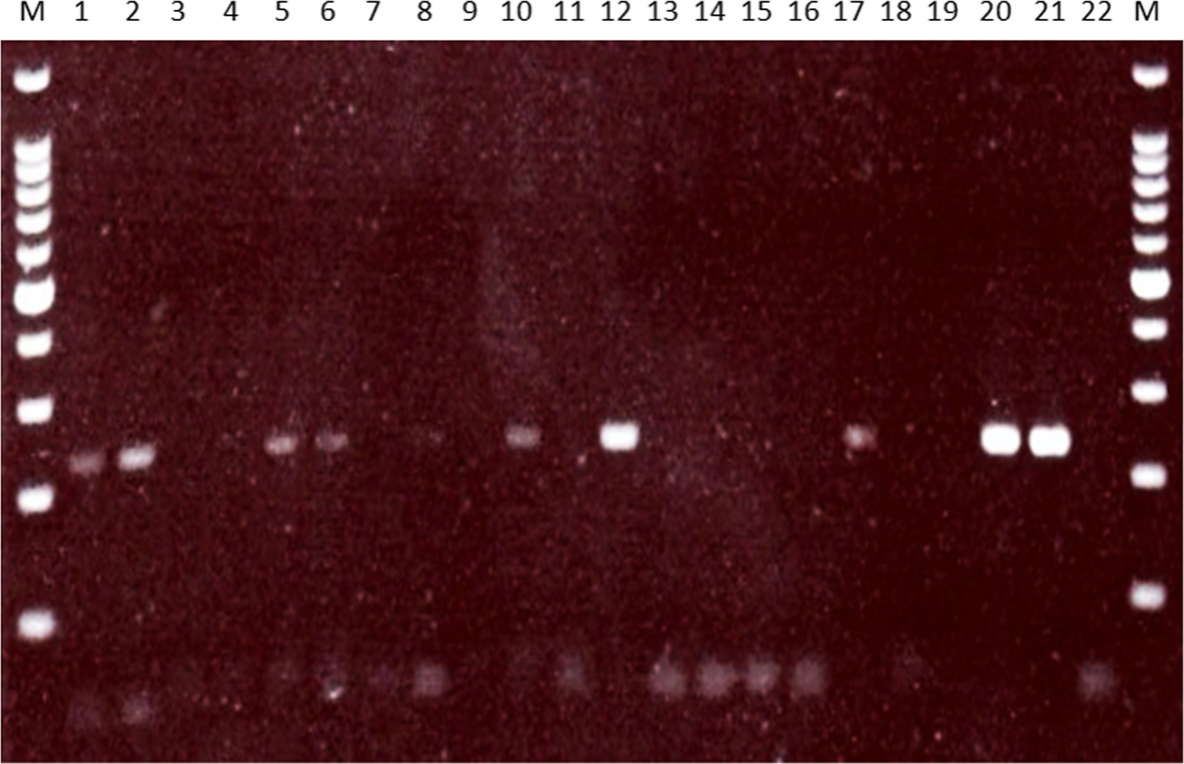
Gel image showing myxoma virus amplification products in mosquito blood-meal samples. The lane order is: 100 bp ladder (M), blood-fed *Anopheles atroparvus* DNA extracts BF1 (1), BF13 (2), BF14 (3), BF19 (4), BF20 (5), BF31 (6), BF47 (7), BF33 (8), BF39 (9), BF9 (10), BF85 (11), BF93 (12), BF99 (13), BF106 (14), BF108 (15), BF110 (16), BF111 (17), BF18 (18), 113 (19), myxoma virus positive controls (20, 21), negative control (22)

Rabbits at the collection site had been observed showing facial lesions suggestive of myxomatosis (Fig. 3). All 33 specimens of *An. atroparvus* that were found to have fed on rabbits were tested for the presence of the myxoma virus. In total, amplicons were obtained from nine blood-meal samples (27 %) (Fig. 4). DNA sequencing confirmed that the amplicon was derived from the myxoma virus showing 100 % sequence identity to previously characterised virus genomes from England (GenBank accession number KC660084 [32]).

## Discussion

This study demonstrates that a single DNA extract from the abdomen of a blood-fed mosquito can be successfully used for three purposes in a sequential workflow: (1) for the identification of the vertebrate origin of a blood-meal by sequencing of a 685 bp region of the COI gene, followed by (2) the molecular identification of members of *An. maculipennis* s.l. by sequencing of a 435 bp region of ITS-2, then (3) the detection of a pathogen, in this case the myxoma virus. This approach provided definitive evidence that *An. atroparvus* feeds extensively on both healthy and myxoma-infected rabbits, thus indicating that this mosquito could play a more significant role in the transmission of myxomatosis in wild rabbit populations in the UK than was previously suspected.

Early studies on the feeding behaviour of *An. atroparvus* [18, 22] were limited to morphological identification of specimens. Despite suggestions as far back as the 1930s of a third member of the *An. maculipennis* s.l. in the UK [33], it was not until recent sequencing of ITS-2 was performed that a third member, *An. daciae*, was discovered [7]. Applying current molecular techniques to delineate mosquito species complexes is important as morphologically indistinguishable sibling species may exhibit different feeding behaviours that will affect their capacity to act as disease vectors. Owing to its recent discovery in the UK the feeding preferences of *An. daciae* have not been extensively studied, however one study indicated it may differ from *An. atroparvus* and *An. messeae* by feeding on birds as well mammals [34]. In the present study no *An. daciae* were found but *An. atroparvus* and *An. messeae* were successfully identified by amplification of ITS-2 [29]. The presence of both species in the same collection site is to be expected as Elmley sits at the interface between the saline coastal habitats generally favoured by *An. atroparvus* and freshwater breeding habitats in the grazing marshes more suited to *An. messeae* (see [35] for a review). Both these species were found to feed only on mammals, corresponding with previous studies [34]. However, 92 % of *An. atroparvus* blood-meals were taken from rabbits, whereas *An. messeae* was found to have fed only on cattle (86 %) and dog (14 %). It is worthy of note that although the 14 % of *An. messeae* comprises only a single mosquito, only one dog is present on site (this belongs to the site owners; other dogs are not permitted) and thus the host abundance of this species is considerably lower than that of rabbits around the collection site. Nonetheless it is evident that further blood-fed collections from the site are required to draw conclusions on whether these results reflect a true difference in feeding preferences between the two mosquito species. Prior to this study, there have been no published reports of rabbit blood being detected in field-caught specimens of *An. atroparvus* in the UK (Table 1). The molecular approach used in this study facilitated the finding of this result as sequencing of the ‘universal’ barcoding region of the COI gene precludes the need to pre-select hosts on which mosquito feeding is considered most likely, a necessary step in the preparation of species-specific sera for serological assays such as the precipitin test or when designing primers for a multiplex assay. The previous study collecting *An. maculipennis* s.l. from a similar area of Kent utilised a multiplex assay design that did not include rabbit and therefore was not able to inform on rabbit-feeding behaviour [34].

The apparent strong feeding preference of *An. atroparvus* for rabbits is somewhat surprising considering the low numbers captured previously in a study employing direct capture of mosquitoes, rabbit-baited traps and precipitin testing in the UK [19]. There, only two individuals of the *An. maculipennis* s.l. were collected, both of which were negative for rabbit blood by precipitin testing and neither mosquito were identified to species. A recent host preference study in Spain provided evidence that *An. atroparvus* populations did feed on rabbits in the wild, albeit at a frequency of less than 2 % (2/115 blood-meals) [28]. This contrasts with observations of strong feeding preferences for rabbits by *An. atroparvus* elsewhere in Europe [36], raising the question of whether different populations of the same species may exhibit different feeding preferences driven mainly by the local availability of hosts rather than an intrinsic preference [37]. Collecting larger numbers of blood-fed *An. maculipennis* s.l. from several sites across the UK taking into account local rabbit abundance may provide evidence to this effect. Elmley Nature Reserve is a grazing marsh with cattle present close to the area of collection and throughout the collection period. It would appear that rabbits are being preferentially selected for feeding by *An. atroparvus* despite the presence of larger mammalian hosts, although host-baited choice experiments in the field or laboratory would provide stronger evidence for this behaviour (reviewed in [4]).

It is important to maximise the data that can be obtained from a single blood-fed specimen as collecting large numbers of blood-fed mosquitoes in the UK by currently available methods is challenging [38]. Furthermore, the likelihood of successfully identifying blood-meal host decreases rapidly with time as digestion within the insect takes place (reviewed in [4]). The primer cocktail used in this study [27] successfully identified the blood-meal host in 46 % of the captured blood-fed mosquitoes. The time between feeding and collection was not known for mosquitoes in this study but assessing mosquitoes in future studies using the Sella score to rate level of digestion would be beneficial to assess the efficacy of this approach according to the degree of digestion [39].

The concomitant identification of both the mosquito and its blood-meal host allowed for the targeted selection of specimens of epidemiological relevance for subsequent pathogen screening, saving both time and resources. In this instance we have screened samples for a mechanically transmitted pathogen. Prior to this study, there was little evidence from fieldwork studies that mosquitoes played an important role in the transmission of myxomatosis among wild rabbit populations in the UK. Evidence for occasional involvement in transmission to and within domestic rabbit populations has been reported [18] supported by laboratory transmission data [22]. This study found that 27 % of blood-meals derived from rabbit were positive for myxoma virus. According to evidence that arthropod transmission is mechanical and therefore requires the presence of a lesion through which mouthpart contamination will occur [15], this finding demonstrates that wild, myxomatosis-infected rabbits are being fed upon by *An. atroparvus* at Elmley. However, it is the observation that over two-thirds of rabbit-derived blood-meals were negative for myxoma virus that is most important and differs from previous studies in the UK as this suggests that *An. atroparvus* is not simply opportunistically feeding on diseased rabbits, but also readily feeds on healthy rabbits in wild populations. We therefore conclude that mosquitoes could contribute to the transmission of the virus. Mosquito collections in this study were conducted between June and September, the period broadly considered to be the peak vector season in the UK [21, 40], however, the number of mosquitoes collected make interpreting seasonal influences uncertain. If the peak incidence of myxomatosis in the rabbit population were to be closely associated with the peak period of *An. atroparvus* activity, then this would provide evidence of seasonal shifts in the relative importance of different vector groups in the area.

The myxoma virus possesses a DNA genome and was therefore present following the extraction procedure. However, using a DNA- or RNA-specific extraction protocol provides an additional limitation to the data that can be obtained from each specimen. Therefore, we advocate the use of a co-extraction procedure such as that of Griffiths [41] that would preserve both DNA and RNA, greatly widening the information that could be obtained from a single mosquito specimen. Further applications of such an approach could include screening for human viral pathogens present in mosquitoes that had fed on humans for the purposes of xenosurveillance [42].

## Conclusions

This study shows that a single DNA extraction can be successfully used to identify blood-meal host, delineate to species level members of the *An. maculipennis* s.l. and to subsequently detect the presence of an animal pathogen, myxoma virus. This tripartite approach revealed that healthy and myxomatosis-infected rabbits are a major blood-feeding host at Elmley, Kent, and therefore provides further evidence that *An. atroparvus* may play an important role in the transmission of this disease in wild rabbit populations.

## References

[1] Mukabana WR, Takken W, Knols BGJ (2002). Analysis of arthropod bloodmeals using molecular genetic markers. Trends Parasitol.

[2] Benson DA, Karsch-Mizrachi I, Lipman DJ, Ostell J, Wheeler DL (2005). GenBank. Nucleic Acids Res.

[3] Ratnasingham S, Hebert PDN (2007). bold: The Barcode of Life Data System (http://www.barcodinglife.org). Mol Ecol Notes.

[4] Kent RJ (2009). Molecular methods for arthropod bloodmeal identification and applications to ecological and vector-borne disease studies. Mol Ecol Resour.

[5] Muñoz J, Ruiz S, Sorigeur R, Alcaide M, Viana DS, Roiz D, Vázquez A, Figuerola J (2012). Feeding patterns of potential West Nile virus vectors in south-west Spain. PLoS One.

[6] Prakash A, Walton C, Bhattacharyya DR, Loughlin SO, Mohapatra PK, Mahanta J (2006). Molecular characterization and species identification of the Anopheles dirus and An. minimus complexes in north-east India using r-DNA ITS-2. Acta Trop.

[7] Linton Y, Lee A, Curtis C (2005). Discovery of a third member of the Maculipennis group in SW England. Eur Mosq Bull.

[8] Takken W, Verhulst NO (2013). Host preferences of blood-feeding mosquitoes. Annu Rev Entomol.

[9] Kerr PJ, Best SM (1998). Myxoma virus in rabbits. Rev Sci Tech.

[10] Aragão HB (1943). O virus do mixoma no coelho do mato (Sylvilagus minenses), sua transmissão pelos Aedes scapularis e aegypti. Mem Inst Oswaldo Cruz.

[11] Armour CJ, Thompson HV (1955). Spread of myxomatosis in the first outbreak in Great Britain. Ann Appl Biol.

[12] Hudson JR, Thompson HV, Mansi W (1955). Myxoma Virus in Britain. Nature.

[13] Ross J, Sanders MF (1984). The development of genetic resistance to myxomatosis in wild rabbits in Britain. J Hyg (Lond).

[14] Lockley R (1954). The European rabbit flea, Spilopsyllus cuniculi, as a vector of myxomatosis in Britain. Vet Rec.

[15] Fenner F, Woodroofe GM (1953). The pathogenesis of infectious myxomatosis; the mechanism of infection and the immunological response in the European rabbit (Oryctolagus cuniculus). Br J Exp Pathol.

[16] Foley DH, Wilkerson RC, Cooper RD, Volovsek ME, Bryan JH (2007). A molecular phylogeny of Anopheles annulipes (Diptera: Culicidae) sensu lato: the most species-rich anopheline complex. Mol Phylogenet Evol.

[17] Sobey WR, Conolly D (1971). Myxomatosis: the introduction of the European rabbit flea Spilopsyllus cuniculi (Dale) into wild rabbit populations in Australia. J Hyg (Lond).

[18] Muirhead-Thomson RC (1956). Field studies of the role of Anopheles atroparvus in the transmission of myxomatosis in England. J Hyg (Lond).

[19] Service MW (1971). A reappraisal of the role of mosquitoes in the transmission of myxomatosis in Britain. J Hyg (Lond).

[20] Service MW (1971). Feeding behaviour and host preferences of British mosquitoes. Bull Entomol Res.

[21] Service MW (1969). Observations on the ecology of some British mosquitoes. Bull Entomol Res.

[22] Andrewes CH, Muirhead-Thompson RC, Stevenson JP (1956). Laboratory studies of Anopheles atroparvus in relation to myxomatosis. J Hyg (Lond).

[23] Muirhead-Thomson RC (1956). The part played by woodland mosquitoes of the genus Aedes in the transmission of myxomatosis in England. J Hyg (Lond).

[24] Panella NA, Crockett RJ, Biggerstaff BJ, Komar N (2011). The Centers for Disease Control and Prevention Resting Trap: a novel device for collecting resting mosquitoes. J Am Mosq Control Assoc.

[25] Snow KR (1990). Mosquitoes. Naturalists’ Handbook 14.

[26] Messing J (1983). New M13 vectors for cloning. Methods Enzymol.

[27] Ivanova NV, Zemlak TS, Hanner RH, Hebert PDN (2007). Universal primer cocktails for fish DNA barcoding. Mol Ecol Notes.

[28] Martínez-de la Puente J, Ruiz S, Soriguer R, Figuerola J (2013). Effect of blood meal digestion and DNA extraction protocol on the success of blood meal source determination in the malaria vector Anopheles atroparvus. Malar J.

[29] Collins FH, Paskewitz SM (1996). A review of the use of ribosomal DNA (rDNA) to differentiate among cryptic Anopheles species. Insect Mol Biol.

[30] Li Y, Meyer H, Zhao H, Damon IK (2010). GC content-based pan-pox universal PCR assays for poxvirus detection. J Clin Microbiol.

[31] Belsham GJ, Polacek C, Breum SØ, Larsen LE, Bøtner A (2010). Detection of myxoma viruses encoding a defective M135R gene from clinical cases of myxomatosis; possible implications for the role of the M135R protein as a virulence factor. Virol J.

[32] Kerr PJ, Rogers MB, Fitch A, Depasse JV, Cattadori IM, Twaddle AC, Hudson PJ, Tscharke DC, Read AF, Holmes EC, Ghedin E (2013). Genome scale evolution of myxoma virus reveals host-pathogen adaptation and rapid geographic spread. J Virol.

[33] Edwards FW (1936). Probable occurrence in England of the so-called typical race of Anopheles maculipennis. Entomologist.

[34] Danabalan R, Monaghan MT, Ponsonby DJ, Linton Y-M (2014). Occurrence and host preferences of Anopheles maculipennis group mosquitoes in England and Wales. Med Vet Entomol.

[35] Sinka ME, Bangs MJ, Manguin S, Coetzee M, Mbogo CM, Hemingway J, Patil AP, Temperley WH, Gething PW, Kabaria CW, Okara RM, Van Boeckel T, Godfray HCJ, Harbach RE, Hay SI (2010). The dominant Anopheles vectors of human malaria in Africa, Europe and the Middle East: occurrence data, distribution maps and bionomic précis. Parasit Vectors.

[36] Cambournac FJ (1994). Contribution to the history of malaria epidemiology and control in Portugal and some other places. Parassitologia.

[37] Chaves LF, Harrington LC, Keogh CL, Nguyen AM, Kitron UD (2010). Blood feeding patterns of mosquitoes: random or structured?. Front Zool.

[38] Burkot TR, Russell TL, Reimer LJ, Bugoro H, Beebe NW, Cooper RD, Sukawati S, Collins FH, Lobo NF (2013). Barrier screens: a method to sample blood-fed and host-seeking exophilic mosquitoes. Malar J.

[39] Detinova TS (1962). Age-grouping methods in Diptera of medical importance with special reference to some vectors of malaria. Monogr Ser World Health Organ.

[40] Brugman V, Horton D, Phipps LP, Johnson N, Cook AJC, Fooks AR (2013). Epidemiological perspectives on West Nile virus surveillance in wild birds in Great Britain. Epidemiol Infect.

[41] Griffiths RI, Whiteley AS, O’Donnell AG, Bailey MJ (2000). Rapid method for coextraction of DNA and RNA from natural environments for analysis of ribosomal DNA- and rRNA-based microbial community composition. Appl Environ Microbiol.

[42] Grubaugh ND, Sharma S, Krajacich BJ, Fakoli Iii LS, Bolay FK, Diclaro Ii JW, Johnson WE, Ebel GD, Foy BD, Brackney DE (2015). Xenosurveillance: a novel mosquito-based approach for examining the human-pathogen landscape. PLoS Negl Trop Dis.

[43] Service MW (1969). The use of traps in sampling mosquito populations. Entomol Exp Appl.

